# Health professionals’ identified barriers to trans health care: a qualitative interview study

**DOI:** 10.3399/BJGP.2021.0179

**Published:** 2021-10-19

**Authors:** Magdalena Mikulak, Sara Ryan, Richard Ma, Sam Martin, Jay Stewart, Sarah Davidson, Melissa Stepney

**Affiliations:** Department of Social Care and Social Work, Manchester Metropolitan University, Manchester; honorary researcher fellow, Nuffield Department of Primary Care Health Sciences, University of Oxford, Oxford.; Department of Social Care and Social Work, Manchester Metropolitan University, Manchester.; Department of Primary Care and Public Health, School of Public Health, Imperial College London, London.; Nuffield Department of Primary Care Health Sciences, University of Oxford, Oxford.; Voluntary Action Islington, London.; Gender Identity Development Services, The Tavistock and Portman NHS Foundation Trust, London; head of psychosocial and mental health, British Red Cross, London.; Nuffield Department of Primary Care Health Sciences, University of Oxford, Oxford.

**Keywords:** general practice, gender identity, health services for transgender persons, delivery of health care, qualitative research, primary health care

## Abstract

**Background:**

Trans and gender-diverse people face multiple barriers within health care. Primary care practitioners are key to providing health care to trans and gender-diverse people but they often lack training in, and understanding of, trans identities and healthcare options. Few studies have examined health professionals’ understanding of the barriers that exist in health care for trans and gender-diverse people.

**Aim:**

To map out barriers to providing good-quality health care to trans and gender-diverse people, and explore ways to address them.

**Design and setting:**

A qualitative interview study involving 20 health professionals working with young trans and gender-diverse people.

**Method:**

Participants were recruited through purposive and snowball sampling. Data were generated using semi-structured qualitative interviews. A thematic analysis involved coding and categorising data using NVivo (version 12) software and further conceptual analysis in which developing themes were identified.

**Results:**

Four barrier domains to good-quality care for trans and gender-diverse people were identified: structural (related to lack of guidelines, long waiting times, and shortage of specialist centres); educational (based on lack of training on trans health); cultural and social (reflecting negative attitudes towards trans people); and technical (related to information systems and technology).

**Conclusion:**

There is an urgent need to address the barriers trans and gender-diverse people face in health care. Structural-level solutions include health policy, professional education, and standards; at the practice level, GPs can act as potential drivers of change in addressing the cultural and technical barriers to better meet the needs of their trans and gender-diverse patients.

## INTRODUCTION

Trans and gender-diverse people in the UK have varied and distinct health and care needs.^1^^–^2 Trans is used here as an abbreviation of transgender to denote a person who feels their sex/gender assigned at birth does not match their sense of self. Gender diversity encompasses people who express their gender in diverse ways, including those not conforming to societal norms and binary gender expectations.

There are several avenues through which trans people gain access to specialist health care; however, the current established entry point remains through primary care and being referred to specialist services by a GP.^2^ In the UK, the NHS provides specialist trans health care for adults and children. In England, adult specialist care is provided by one of seven gender identity clinics. For those aged <18 years, care is provided by the gender identity development service.

Seeking health care as a trans person is often described as a challenging and frustrating process.^3^ Disparities that trans people face in health care have been linked to health professionals’ lack of knowledge regarding trans health as well as negative attitudes towards trans people.^4^^–^6 Recently, the UK House of Commons Women and Equalities Committee reported lack of knowledge and understanding, and in some cases anti-trans prejudice, as barriers to trans people receiving care.^7^

Research shows that a lack of knowledge on the part of health professionals can also contribute to them feeling ‘uncomfortable’ when working with trans patients.^8^ On the patients’ side, negative experiences with health professionals can lead to trans and gender-diverse people avoiding seeking general and sexual health care,^9^ anticipating that providers *‘would not only be unprepared to meet their medical needs, but may also be unprepared for their very existence’*.^10^ Prejudice and discrimination in healthcare settings can take different forms including but not limited to withholding or denying services, misgendering patients, and inappropriate questioning.^1^^–^2

Trans health care is further complicated by existing disciplinary divisions of responsibilities within medicine, with few health professionals identifying trans health care as an interest area.^6^ However, evidence suggests that the addition of a simple curricula change in medical school to include trans-related care can improve students’ knowledge and attitudes towards trans patients.^8^

**Table:** How this fits in

Trans and gender-diverse people face a range of barriers within health care, which signals that health professionals, including primary care practitioners, are often not able to effectively support them. Structurally, a shortage of specialist services and long waiting times are major impediments but other barriers exist that complicate access to care and exacerbate health inequalities for this patient group. While providing an overview of structural barriers that trans and gender-diverse people face, this study identifies educational, social and cultural, and technical barriers to providing health care for these patients. It makes recommendations to strengthen primary care practitioners’ ability to better meet the needs of this underserved population.

It is clear from this evidence that the barriers encountered by trans and gender-diverse people in health care are varied and often multiple, which can have an impact on healthcare access and use.^3^ Currently, there is a lack of mapping of these barriers in a UK context, including health professionals’ own understanding of these. The aim of this study was to provide such a mapping, with particular focus on primary care, to better identify and address these barriers.

## METHOD

This study draws on in-depth qualitative interviews with 20 health professionals (see Box 1 for participant characteristics) based in the UK conducted as part of a larger research project on the health experiences of young trans people. Although the interviews were with health professionals working with young adults, the authors did not limit the responses to discussion of caring for young people and the findings from this study could be applied to trans and gender-diverse people of all ages.

**Box 1.** Participants

**Participant ID**	**Profession**	**Gender**
HP1	GP	Cisgender male
HP2	GP	Cisgender female
HP3	Oncologist	Cisgender male
HP4	Practice nurse	Cisgender female
HP5	GP	Cisgender female
HP6	GP	Trans female
HP7	Counselling professional	Trans female
HP8	GP	Cisgender female
HP9	Psychologist	Cisgender female
HP10	GP	Cisgender female
HP11	Mental health practitioner	Cisgender female
HP12	Psychiatrist	Cisgender male
HP13	Psychologist	Cisgender female
HP14	Counselling professional	Non-binary
HP15	Psychiatrist	Cisgender female
HP16	Specialist registrar	Cisgender female
HP17	GP	Trans female
HP18	Voice therapist	Cisgender female
HP19	Endocrinologist	Cisgender male
HP20	GP	Cisgender female

*HP = health professional.*

### Sampling and recruitment

A broad range of health professionals were recruited to the study, including GPs, practice nurses, counsellors, and mental health practitioners. Participants were selected purposefully on the basis of their experience of working with young trans and gender-diverse people. Purposeful sampling seeks to understand groups, settings, and individuals where the processes studied are most likely to occur.^11^ It demands that researchers think critically about the parameters of the population they are studying.^11^ Participants were approached through co-applicant networks, social media routes, advisory group members, local and national support groups, and snowballing through personal contacts and research participants. Recruitment materials (online posters and leaflets) with ethical approval were advertised through these channels.

### Data collection

The semi-structured telephone interviews were conducted between June 2019 and February 2020 by two researchers. Interviews used methods that had been approved for national studies by the National Research Ethics Service Committee South Central — Berkshire.

A topic guide (a series of questions formulated to guide the interviewer; see Supplementary Appendix S1) was developed from a synthesis of the literature using a range of health and social science databases, and subsequently amended to include new questions emerging from the ongoing analysis. Interviews were on average approximately 90 min and allowed participants to talk about their role and experiences of working with trans and gender-diverse people, express their views about the current healthcare pathway, and suggest service improvements. The interviews were audiorecorded and transcribed non-verbatim by a professional transcriber. The participants were asked to validate the transcripts.

### Analysis

An analytical approach was taken that combined thematic analysis and modified grounded theory.^12^^–^13 A staged analysis began with initial reading and re-reading of the data. Data analysis was an iterative process with data collection, coding, and analysis proceeding in tandem. NVivo (version 12) analysis software was used to organise, code, and categorise the data. Data were constantly compared, in terms of initial codes and the subsequent identification and development of concepts/themes.

The coding was undertaken independently by the first author and was checked and compared by the second and fourth authors. This was followed by a more conceptual analysis focusing on a key theme identified through the analytical process: barriers to health care.

## RESULTS

The identified barriers to health care can be mapped onto four key domains: structural, educational, cultural and social, and technical.

### Structural barriers

Issues identified around shortage of services, long waiting times, lack of guidelines, and lack of funding and support emerged as important themes and have been grouped here as structural barriers.

Many participants expressed dismay about structural barriers that compromised the quality of care for trans and gender-diverse people. Shortage of clinics was identified as a key issue. As one GP said:

*‘There* [are] *so few gender identity clinics around in this country … They simply cannot cope with the demand of the trans patients.’*

(Health professional [HP]6, GP)

The resulting waiting times to access the specialist services were also identified as a major barrier. Some described it as at odds with the NHS commitment to provide timely access to specialist services:

*‘The patient’s charter says 18-weeks wait in England … we have now got to the stage where my local gender clinic is two and a half years wait.’*

(HP14, counselling professional)

Discussions around long waiting lists were placed in the wider context of waiting lists across the NHS, and delay in accessing mental health services were identified as an additional barrier to supporting trans and gender-diverse people.

Further, lack of support and access to clear clinical guidelines for care pathways and treatment were identified as key challenges. One GP said:

*‘There seems to be a big void out there for managing these patients. It’s all very much back to primary care. We can’t do anything. We are not allowed to initiate medications … We don’t have the back-up. We don’t have numbers that we can ring and say, “help what would you do in this situation, please?” or “what resources could we access?” There is just nothing there.’*

(HP20, GP)

Participants raised questions about the lack of guidelines and one GP described her failed attempt to get more guidance:

*‘I wrote to NICE* [National Institute for Health and Care Excellence] *… and I said, they have guidelines on everything … have you got any guidelines on the treatment of transgender people, “no we haven’t and we haven’t got any plans to do in the immediate future.”’*

(HP5, GP)

Participants also emphasised that they were working with trans and gender-diverse people without clear guidance from local clinical commissioning groups.

Health professionals also commented on the lack of support for patients while waiting for specialist services. One therapist commented:

*‘You are going completely unsupported by any medical professional when you are experiencing very significant mental distress.’*

(HP14, counsellor)

In addition, participants recognised how huge waiting times pushed trans and gender-diverse people to use private care services, but that this meant there was lack of support for GPs in managing any resulting shared care agreements.

### Educational barriers

The next key domain was the lack of education and training in trans health. One GP thought this was a particularly pressing issue for primary care:

*‘The first port of call* [for trans people] *is … their GP. And so I would say that that’s the first barrier, actually … I think that is the biggest barrier there is … GP education.’*

(HP2, GP)

All participants thought that trans health was not sufficiently covered by their education and professional training:

*‘I went through medical school … there was nothing that I can remember at all on trans identities and health care for trans people.’*

(HP3, oncologist)

A GP observed:

*‘In medical school there was no information or education* [on trans health] *... When I was training to be a GP there was none.’*

(HP5, GP)

A practice nurse made a similar point:

*‘Whilst I was training to become a nurse there was absolutely nothing about transgender health care.’*

(HP4, practice nurse)

Part of educational barriers was not being aware of local resources to signpost patients to. A GP stated:

*‘If you don’t know the resources locally then you cannot signpost people to the right places and so in, in our area we’ve got some fantastic community resources. But, you know, in my practice probably maybe three or four of us know them and would be able to signpost.’*

(HP17, GP)

### Cultural and social barriers

Interrelated with a lack of knowledge of trans and gender-diverse identities and trans health, negative attitudes towards trans people were identified as a key barrier in primary care. One GP noted:

*‘Some GPs will be actively against it and say that trans is not a real thing and the NHS shouldn’t be funding trans care.’*

(HP6, GP)

A counsellor remarked how negative attitudes create discomfort:

*‘I don’t think they* [GPs] *really know how to deal with their own prejudice around this [trans healthcare] and that it’s making them face something that they feel uncomfortable with.’*

(HP7, counsellor)

Some participants felt that non-binary gender identities posed a particular challenge for health professionals:

*‘I think it becomes sort of difficult for sort of health professionals to understand sometimes the non-binary. I think because it’s easier for health professionals to think in [terms of] masculine/feminine.’*

(HP1, GP)

A therapist, who identified as non-binary, thought that negative attitudes more generally reflected conservative ideas about gender in society:

*‘I think there are still a lot of misunderstanding around non-binary issues and often kind of conservative ideas about gender [as binary] .’*

(HP14, therapist)

Most participants identified challenges related to their and others’ communication, with language around trans and gender-diverse identities, pronouns, and titles, and lack of cultural competency — which links back to educational barriers — around these as an issue affecting the care provided to trans and gender-diverse people. One GP observed:

*‘I think that terminology and language is poor. I think GPs grapple and struggle just to really understand conversations around* [gender] *identity …* [including the] *use of pronouns.’*

(HP2, GP)

The use of singular ‘they’ was identified as a particular challenge:

*‘I think the most difficult for professional people, I think is them/their/theirs because it goes, I suppose it goes against your English education.’*

(HP11, mental health practitioner)

### Technical barriers

Issues with not using the correct gender and pronouns were amplified by technical barriers; in particular, inflexible computer systems with no options to record the gender a person identifies with as well as their natal one. One GP stated:

*‘I did an audit of a cohort of trans people. When it came to whether their name on the clinical system and their gender marker on the clinical system reflected their gender marker and name in real life. Then only 50% of them had alignment.’*

(HP17, GP)

Inflexible computer systems can lead to both misgendering and exclusion from necessary check-ups and scans, with smear tests for people who have cervixes used as a common example:

*‘After a patient has transitioned … because their name and their gender changes on the system records. They don’t get alerts* [...] *understandably, they don’t want to acknowledge that they might need a smear.’*

(HP20, GP)

Display systems in waiting rooms and reception booking systems were identified as another technical barrier that complicated the use of people’s names, titles, and pronouns:

*‘Within sort of health systems … we do have their birth name on our systems until they change it, you know, which can make it more difficult … with sort of … reception and things like that.’*

(HP11, mental health practitioner)

Physical spaces, such as waiting areas in GP surgeries and reception areas, were also discussed as important to how trans and gender-diverse people felt. Some thought such spaces were not set up to accommodate trans and gender-diverse people as they often were designed around a binary understanding of gender or were not seen as welcoming. Health professionals also raised issues of single sex toilets and hospital wards as being problematic.

## DISCUSSION

### Summary

This study outlines some of the key barriers in primary care for trans and gender-diverse people as identified by healthcare professionals. Participant identified barriers were categorised into four domains that may prevent effective health care for trans and gender-diverse people: structural, educational, cultural and social, and technical. Structural barriers include scarcity of specialist centres and participants expressed a clear desire for decreased waiting times. Lack of clear guidelines was also listed as a significant barrier.

Educational barriers were also a key concern, as all participants stressed that the lack of inclusion of trans health in their training had left them unprepared to care for this population.

The next domain was related to cultural competence and negative attitudes against trans people resulting in discrimination within primary care settings. Issues of language and awareness of trans and gender-diverse identities were also highlighted. The final domain identified technical barriers such as inflexible information technology systems and gendered spaces.

### Strengths and limitations

The study team included individuals who were cisgender, trans, and non-binary, which is a strength because it offers interpretation of data based on lived experience.

All participants interviewed had worked with trans and gender-diverse people, and had either a special interest in the topic or wanted to share their concerns around trans health care. As a result, these were healthcare professionals who were mostly affirmative and supportive of trans care. Better understanding of those health professionals with no knowledge of trans health care or no experience with trans patients would warrant further research.

The diversity of the sample, which included a range of healthcare professionals, meant there was wide variability in participants’ experiences. This, however, left some areas of health care underexplored. The issues highlighted were more heavily focused on primary care settings. A broader study, which concentrates on key groups in secondary care within this sample (such as endocrinologists), might provide further in-depth information. The findings in the current study have potential applicability to different settings with trans people of all ages; for example, mental health services.

More focused research might reveal further insights around general health care after medical transition, health screening and risk assessments, sexually transmitted disease checks, cervical cytology, pregnancy, cardiovascular risk management, and possibly other areas.

### Comparison with existing literature

The findings confirm issues identified in existing literature but also add new insights on the complexity and multitude of barriers trans and gender-diverse people encounter in health care.

Access, lack of support, and education are key factors that have an impact on trans health care.^6^ Findings on prejudice and lack of cultural competence in working with trans and gender-diverse people echo previous studies suggesting that more needs to be done to counter negative attitudes and ignorance within health care.^10^ This study also identifies technical barriers as an additional area, which compounds and exacerbates challenges for trans and gender-diverse people.

The World Professional Association for Transgender Health has established internationally accepted guidelines for care and treatment of transgender individuals.^14^ In addition to providing general care to trans and gender-diverse people, primary care practitioners play a key role in facilitating specialist trans health care. The authors are aware of three new pilot schemes in England (in London, Merseyside, and Manchester) being trialled using a patient-centred informed consent model for transition, moving more care into general practice. An international study has already planned to investigate the role of primary care in trans and gender-diverse people.^15^ To the authors’ knowledge, the previous lack of UK-based studies on health professionals’ experiences of trans health care means that the findings in this study around structural and educational barriers offer new insights into how to improve trans health care as it changes and evolves. At the same time, findings on educational barriers reverberate issues identified in other countries pointing to the need for more focused training, which has proven effective in other contexts.^16^^–^17 Similarly, identified barriers around inflexible computer systems highlight an area that needs addressing within healthcare settings to better reflect the experiences and needs of trans and gender-diverse people.^18^

### Implications for research and practice

The four barrier domains overlap and compound issues of access and service use for trans and gender-diverse people. It is therefore key that these are addressed simultaneously to improve trans health care and primary care for trans and gender-diverse people.

Structural barriers necessitate wide scale improvement, such as an expansion of services within this area, whereas cultural barriers can be somewhat addressed by the inclusion of trans health in medical curricula and training. Heightened awareness of the needs of this patient group would enable a better understanding of technical barriers that can be tackled systemically and at the local level in individual practices.

Moreover, exploring the structural, cultural and social, and educational barriers facing trans patients has further resonance for other minority groups experiencing health disparities. The domains identified parallel the multilevel and intersecting inequalities faced by other socially disadvantaged groups — of which trans and gender-diverse people can also be members — including those who experience racial discrimination, sexual minorities, and people with disabilities.^19^^–^21

Further research is required to identify the specific challenges faced by primary care practitioners at different stages of caring for trans and gender-diverse people, from issues with the referral process to prescribing and shared care arrangements. More research is also needed to understand the specific educational needs of primary care practitioners in relation to trans health. The data suggests training is needed to challenge negative attitudes and to provide better understanding of gender diversity, as well as to make sure primary care practitioners are familiar with the existing pathways for trans youth and adults.

Given the lack of research in this area, mixed-population studies would be valuable to study both health professionals and patients’ perspectives. These findings can then be applied to improve the processes that bring trans health care more successfully within primary care settings. In addition to providing general care to trans and gender-diverse people, primary care practitioners play a key role in facilitating specialist trans health care. More resources and efforts are needed to ensure that GPs are well equipped and supported in their work with this underserved population. Potential solutions include clear clinical guidelines and commissioning pathways, as well as inclusion of trans health in education and training. Although structural and educational barriers need to be addressed top down, with better training and support, GPs can act as potential drivers of change in addressing the cultural and social, and technical barriers to better meet the needs of trans and gender-diverse people who are in their care.
